# Multisource Assessment for Development Purposes: Revisiting the Methodology of Data Analysis

**DOI:** 10.3389/fpsyg.2018.02646

**Published:** 2019-01-04

**Authors:** Joan Manuel Batista-Foguet, Willem Saris, Richard E. Boyatzis, Ricard Serlavós, Ferran Velasco Moreno

**Affiliations:** ^1^ESADE BS, Universitat Ramon Llull, Barcelona, Spain; ^2^Political Sciences Department, Universitat Pompeu Fabra, Barcelona, Spain; ^3^WSOM, Case Western Reserve University, Cleveland, OH, United States

**Keywords:** multisource assessment, social and emotional competencies, factorial invariance, leadership development, structural equation modeling

## Abstract

Multisource assessment (MSA) is based on the belief that assessments are valid inferences about an individual’s behavior. When used for performance management purposes, convergence of views among raters is important, and therefore testing factor invariance across raters is critical. However, when MSA is used for development purposes, raters usually come from a greater number of contexts, a fact that requires a different data analysis approach. We revisit the MSA data analysis methodology when MSA is used for development, with the aim of improving its effectiveness. First, we argue that having raters from different contexts is an integral element of the assessment, with the trait–context dyad being the actual latent variable. This leads to the specification of an *Aggregate* (instead of the usual *Latent*) multidimensional factor model. Second, since data analysis usually aggregates scores for each rater group into a single mean that is then compared with the self-rating score, we propose that the test for factor invariance must also include scalar invariance, a pre-requisite for mean comparison. To illustrate this methodology we conducted a 360° survey on a sample of over 1100 MBA students enrolled in a leadership development course. Finally, by means of the study we show how the survey can be customized to each rater group to make the MSA process more effective.

## Introduction

One the most commonly used methods of measuring leadership effectiveness is of multisource assessment (MSA), which involves self-assessment and assessments by others (typically bosses, peers and direct reports) of an individual’s behaviors or performance in a particular environment. The central premise underlying MSA is that the focal subject benefits from anonymous feedback from multiple sources (London and Smither, 1995) which is typically delivered in a numerical form, all in an attempt to assure greater objectivity and fairness (Lawler, 1967; Toegel and Conger, 2003).

Multisource assessment can be used either for performance appraisals, mostly referred to as multisource performance ratings (MSPR), or for development purposes, most commonly known as multisource feedback (MSF), or 360° feedback. MSPR is used as an alternative to traditional performance appraisals (Toegel and Conger, 2003), and often as a basis for decision-making processes such as compensation, succession planning, promotion or even downsizing (Bracken et al., 2001). In contrast, MSF is a managerial development tool based on the assessment of competencies by multiple sources. The use of MSF has grown dramatically over the last 25 years mainly fostered by the proliferation of leadership development programs for managers and executives (Toegel and Conger, 2003), due to the growing concern of organizations to develop and retain their talent.

Since Lawler (1967) paper, the literature provides numerous studies proposing a methodology for analyzing multisource data and validating the measurement scales. These studies have mostly been conducted in the workplace to ensure that the ratings using MSA performance management (MSPR) provide valid inferences. However, we argue that when MSA is used for providing feedback (MSF), that is, for development purposes, the context constitutes a totally different paradigm and therefore we cannot mimic the methodology used for MSPR. Lacking a proper methodology that helps stablishing for construct validity of MSF tools, the quality of the feedback cannot be ensured and hence the effectiveness of the leadership development programs using such tools is in question. The main purpose of this study was therefore to address this problem by proposing a new and specific methodology for designing and identifying construct validity evidence of MSF tools.

The MSF may be illustrated by the Indian fable of six blind men who touched different parts of an elephant and described the animal differently, from the perspective of the part they were touching. One moral we may draw from this fable is the importance of considering all viewpoints to obtain a full picture of reality. Another moral is that all of the blind men were right, in that each correctly described a different part of the elephant.

In performance appraisals (MSPR), accuracy is especially important, as all raters should seek to assess the same performance dimensions, and therefore convergence of ratings is key. However, in MSF a multitude of perspectives, even if they lead to contradictory judgments, can help the rates (clients) to produce a more comprehensive assessment of themselves. For this reason, some MSF also includes feedback from family, friends or customers (Boyatzis et al., 2002). As the Indian fable illustrates, MSF does not need to look for convergences in the raters’ interpretations, since they are complementary rather than concurrent. In this framework, the gap between self-perception and others’ perceptions is key as it provides a valuable source of information for designing a development plan or coaching program.

As previously stated, most studies that discuss the methodology for validating MSA tools have focused on the workplace and therefore have mostly dealt with MSPR. However, when dealing with MSF, adopting the same methodology is inappropriate as it fails to take into account the relevance of comparing self-assessment with each of the multiple perspectives, a comparison that is critical for establishing the development plan.

The new methodology involves a data analysis process that takes into account two differential features of the MSF paradigm: (1) the greater number of contexts that lead to diverse perspectives, and (2) the eventual self-others comparison in the data analysis. This proposed methodology is then illustrated through a study using a 360° survey^1^ based on behavioral emotional intelligence competencies, among a sample of 1194 MBA students enrolled in a leadership development program at a leading European business school. Data were collected over a period of 11 years (from 2006 to 2017).

Our paper presents three main contributions. First, context becomes an integral element of the assessment, since raters have usually different perspectives, and the individual being rated show different behaviors depending on the context. We therefore propose that the trait to be assessed by the measurement model should not be the competency itself but the *competency-context* dyad. Second, data analysis in MSF necessarily involves aggregating scores of all raters in each rater group into a single mean which is then compared with the self-rating score. For this comparison to be valid, we propose that factor invariance, also known as measurement invariance, measurement equivalence, or construct comparability (Meredith, 1993), should include scalar invariance. While this is not pertinent in MSPR, it constitutes a prerequisite for mean comparisons in MSF. Third, we show how the 360° survey can be customized, and hence shortened, to each rater group. These contributions should help academics who do research in MSF to design and validate more effective MSF tools. Shorter and customized MSF surveys should generate higher data quality as well as higher feedback quality, and as a result, participants should be able to draw more appropriate development plans.

In the next section, we revisit the methodology. In the method section, we illustrate the proposed methodology in an empirical study. We then present the results of the study, and finally the discussion and conclusions section elaborates on the aforementioned contributions and presents some suggestions for future research and implications for practice.

## Methodology in MSA

### Flaws in MSA

The MSA’s roots may lie in a reaction to the stage of psychological research during the 1950s and 1960s that was dominated by single measurement methods, which led to highly subjective concepts. Campbell and Fiske (1959, p. 101) claimed that any single operationalization of a concept is equivocal and, to overcome this pervasive single operationalism, they advocated “a multiple operationalism, a convergent operationalism (Garner, 1954), a methodological triangulation, an operational delineation (Campbell, 1954), a convergent validation”.

During the succeeding decades researchers have supported the use of MSPR for appraisal evaluation. In this context, there is evidence showing that ratings from different appraisal ratings are comparable (see Facteau and Craig, 2001; Viswesvaran et al., 2002), although less than it was thought (Viswesvaran et al., 2005). However, some weaknesses have also been pointed out. They stem from biases of the rater source, and others from biases of the individual being rated, i.e., the ratee. Scullen et al. (2000) propose two main classifications of rater biases: those related to idiosyncratic tendencies—the halo effect or leniency—and those related to organizational perspectives. The latter may originate from three sources: (1) differences in raters’ perceptions of available information (Lawler, 1967; Landy and Farr, 1980); (2) focusing attention on different facets of the subject’s performance due to raters coming from different organizational levels (Borman, 1974); and (3) raters’ use of different weighting criteria (Borman, 1974; Atkins and Wood, 2002), as a result of different organizational perspectives giving a different importance to an observed behavior.

Regarding the rater biases that relate to the ratee, these originate in the individual’s “multiple or possible selves” (Ibarra, 1999), as the expression of self may not be independent of the context in which an individual acts. Since individuals behave differently in different contexts, observations of individual behavior vary depending on the nature of the rater-ratee relationship (Lawler, 1967).

Like the blind men in the Indian fable, raters often have different, yet equally valid, partial views of an individual, which may lead to discrepancies in ratings. Several scholars have acknowledged that an honest assessment by different raters could lead to diverse and sometimes contradictory views, such as Borman (1997) and Yammarino (2003) for studies on MSF, or Meredith (1993); Vandenberg and Lance (2000), and Hoffman and Woehr (2009) for studies on MSPR. Other scholars have even argued that rating inconsistency is inherent to MSAs, such as Penny (2001, 2003) for MSF, or Hassan and Rohrbaugh (2009) for MSPR. Accordingly, researchers have tried to shed light on source differences, rating incongruences (Scullen et al., 2003; Hassan and Rohrbaugh, 2009), and information gaps of the specific parties involved (Semeijn et al., 2014).

Rating incongruences were quantitatively analyzed by Scullen et al. (2000) in their analysis of the MSPR dimensional structure. They showed that the rating variance associated with any subject’s performance was lower than the variance associated with rater biases. They found that idiosyncratic variance was the largest component of variance for all combinations of sources and performance dimensions. As a consequence, when assessing MSPR construct validity, Scullen et al. (2003) sensibly do not consider the rater *perspective-related variance* as rater bias, but as rater specificity due to the specific criterion perspective (Tornow, 1993). In other words, rating differences are more a function of true differences in the performance observed by each type of rater in different contexts (Borman, 1974) than of differences in the observers themselves. This is because raters observe different aspects of the ratee’s performance. As Scullen et al. (2000, p. 966) point out, “perspective-related variance should be added to the general and dimensional performance variance to fully account for performance-related variation in ratings.” Regardless of whether rater effect is classified as actual performance or bias, it is always deemed a source of either trait variance or specific variance.

### Flaws in the Data Analysis Method Underpinning MSA

With the emergence of MSA, Campbell and Fiske (1959) argued that the quality of measurement instruments can only be determined by comparing them with other instruments, since estimation of the method effects (the measurement error components) requires repeated measures of the same individuals using different methods. Consequently, ensuring construct validity must involve trait measurement by means of different approaches (e.g., through Multi-Trait Multi-Method, MTMM) (Campbell and Fiske, 1959). In fact, most of the examples Campbell and Fiske (1959) provided to illustrate their MTMM approach were related to MSA and had specified external raters as method factors. In their conclusions, they observed that the amount of variance in the method invalidity significantly exceeded that of trait variance, a finding which exposed the weakness of the validity of external raters.

Multi-Trait Multi-Method designs require that all subjects be measured using each method, i.e., subjects must be fully crossed with methods (Campbell and Fiske, 1959). Thus, MTMM is the appropriate approach for analyzing the structure of the covariance matrix, because it includes all trait–method combinations. Although MSA (both MSPR and MSF) involves multiple raters measuring multiple individuals, such an assessment method cannot match the requirements to be strictly considered a MTMM approach.

In the case of MSPR, data usually come from assessment centers, performance ratings, or structured interviews, and involve multiple rating sources (i.e., methods), such as peers, supervisors or subordinates. Even if all raters are from the workplace, MSPR does not always lead to designs in which individual raters are fully crossed with ratees, as the same raters may not be able to assess each one of the ratees. In order to mitigate the effects of this problem, researchers of MSPR select raters (often two) at random within a data set and treat them in a disaggregate way to make the ratings data appear as if ratees are fully crossed with individual raters. In the case that such raters are fully nested within a rater group (e.g., only subordinate ratings), then Multilevel Confirmatory Factor Analysis (ML-CFA) models would be the appropriate data analysis strategy. This case is, however, rare as raters are often chosen to represent more than one organizational perspective, for example, bosses and direct reports (for an illustrative discussion on the disconnection between the functional form of the CFA model for MSA data analysis and the true underlying structure of the data matrix being fitted, see Putka et al., 2011).

In the case of MSF, which typically assesses competencies rather than performance dimensions, data analysis is even farther from the MTMM ideal. The afore mentioned rater biases (Scullen et al., 2000) are therefore more likely to appear in MSF, due to multiple evaluation contexts and due to greater diversity of rater-ratee relationships, than in MSPR, which is always constrained to the professional context only.

In addition, raters from different contexts are never, and can never, be even partially crossed with ratees: professional and personal environments are necessarily diverse, and specific to each ratee. Therefore, data analysis strategies in MSF are always based on *aggregated* ratings within each contextual group, as opposed to *disaggregated* ratings as usually used in the MSPR context.

In MSF, each trait (competency), is usually operationalized by averaging the scores given to each item for a particular rater group, and then averaging these values for all items attached to the trait. This operationalization yields a trait average score by each external rater group (e.g., average score for competency *A* by the peer group). The gap between each rater group average and the self-evaluation score is then assessed. Engaging the ratees in reflecting on these gaps is one of the main purposes of MSF, as it enhances their self-awareness (Woo et al., 2008), and helps them to discover their critical development needs and to design their development plans.

Scholars have attempted to reconcile differences among raters by requiring higher interclass correlations (ICC) to justify creating composite (i.e., average) scores of the views from several raters, such as peers, subordinates and bosses. In MSF, researchers and practitioners should not look for high levels of correlations among raters in order to legitimize a unique average from all raters. As previously argued, the greatest benefit of MSF is having different perspectives on the subject, which usually results in low correlation among different groups of raters. This common practice in MSF is hardly ever used in MSPR, as the latter most often focus on performance evaluation.

We posit that MSF actually produces a kind of social relativity. Although different groups of raters measure a trait using the same instrument, the trait is modified by the different phenomenological perspectives of different groups of observers. We maintain that the MSF framework, which usually involves different behaviors manifested to different raters, as well as differences in the rater perspectives, often imposes specific *blinders* on raters which may prevent assessment convergence among rater groups, and therefore averaging may result in aggregation errors.

In view of the above, the central questions in using MSF should be: (1) How can the effect of both, the rater perspective and the ratee *multiple possible selves*, be modeled as specific variances separate from the trait variance? (2) When are average comparisons from different contextual perspectives pertinent? (3) How can we assess the degree of pertinence of each perspective in assessing a specific competency?

## Methodology Revisited for MSF

The roots of the methodology we are about to propose for MSF can be found in the two following statements. The first is Bridgman’s (1927, p. 10) assertion that “if we have more than one set of operations, we have more than one concept, and strictly there should be a separate name to correspond to each different set of operations.” The second is Borman’s (1974) warning about looking for agreement between self and external evaluations of the same competency (i.e., convergent validity).

Since Scullen et al. (2003) raised concern about the construct validity of informants’ perspectives in their disaggregated MSPR data analysis, scholars have assessed the invariance of the factor structure among each pair of raters, mostly based on Vandenberg and Lance’s (2000) guidelines, according to which the invariance of the factor structure involves testing for configural invariance, metric invariance, and unique variances invariance. This is the appropriate methodology when the purpose is to interpret the correlations among raters, as is the case of MSPR.

Analogously to the contributions made by several scholars to improve the specific data analysis methodology for the use of MSPR (Scullen et al., 2003; Woehr et al., 2005; Putka et al., 2011), the purpose of this paper is to contribute to an appropriate data analysis methodology for the use of MSF, with the ultimate aim of improving the effectiveness of MSF by customizing context-specific questionnaires and thus achieving higher data quality.

Since a crucial step in MSF is to assess the gap between the self-assessment score and the average score from each rater group on a particular competency, the strategy we propose for data analysis must differ from the one mentioned above in MSPR. This difference in strategy is necessary for two reasons: first, because, unlike MSPR data, MSF data must necessarily involve aggregated ratings; and second, because MSF requires the assurance that all rating sources coincide with the origins of the measurement scales, so that rater group averages can eventually be compared. Consequently, we proceed to answer the first two research questions.

With regards to the first question concerning the effect of both, the rater perspective and the ratee *multiple possible selves*, even in the assessment of a much less ambiguous trait such as performance, Scullen et al. (2003) already addressed the specific variance coming from the rater perspective. They corroborated the primacy of the strong rater (method) effects by finding obvious evidence of invalidity, namely that average heterotrait–monomethod correlations were considerably higher than average monotrait-heteromethod correlations). These findings led them to propose the combination of trait and rater as the latent variable.

In MSF, since we assume that context-driven behaviors and raters’ perspectives should be integral elements of the model, we propose specifying the trait-source *dyad*, which Campbell and Fiske (1959, p. 81) called the “trait-method unit,” as a first-order factor. However, two issues differentiate MSF from the MSPR approach. First, the nature of the competencies in MSF entails a higher degree of ambiguity, and second, the context-driven behaviors and raters’ perspectives are usually much more diverse. This leads us to propose the specification of an alternative model to the one in MSPR for structuring the relationship among the dyads (i.e., an alternative to the hierarchical confirmatory factor analysis model, HCFA). In MSF, we should not consider the trait as a multidimensional *latent* second-order factor model of first-order dyads. Instead, we suggest that only an *aggregate* (Law et al., 1998; Jarvis et al., 2003; Bisbe et al., 2007; Wong et al., 2008) second-order factor model is justified. In this model the different dyads provide complementary information about the latent trait, which for development purposes is more useful than convergent views of the same trait. As the moral of the fable indicates, the different views of the blind men should not be considered as reflective of the same reality. Instead, they should be considered complementary perspectives, and therefore they are all necessary to complete the full picture of the elephant.

Following this *dyad* approach, we propose starting data analysis with the specification of a factor analysis model in which each dyad is considered a different *trait*. Based on this premise, for each competency, results consist of as many traits as the number of operationalizations of that specific competency, which are derived from all the rater groups and the participant’s context.

With regards to the second research question (when are average comparisons between different contextual perspectives pertinent?), it seems reasonable that before making any comparison of latent characteristic scores across evaluators, we must assess the level of measurement equivalence of the dyads. This is particularly vital when the groups have different perspectives from very diverse contexts, as is the case when we have personal and professional rating sources. This is especially the case when these scores are eventually going to be compared with self-evaluations. The level of equivalence will determine whether we are entitled to conduct the intended comparison of group means, which affects what inferences can be made. Comparing group means with self-assessment requires meeting the strong measurement equivalence across all dyads. This involves substituting the MSPR third test (equivalence of unique variances) by the scalar invariance test. We acknowledge that this is “one of the least frequently conducted tests” (Vandenberg and Lance, 2000, p. 38), but it is necessary in the MSF approach.

In the study portrayed in the next section, we illustrate the proposed methodology. First, we show how the *trait-context* dyads should be modeled, and then how the strong factor equivalence should be established and tested in MSF. We also address how we can determine the degree of pertinence of each rater perspective in assessing a specific competency, thus answering the remaining third research question.

Our study is based on a leadership development program that uses MSF on emotional and social intelligence competencies (ESC), currently known as the behavioral approach to assess emotional intelligence (Cherniss and Boyatzis, 2013). ESC are especially suitable to illustrate our methodology for MSF, since most of the competencies can be manifested in a great variety of contexts, and thus lend themselves to be assessed by very different rating contexts (personal and professional). Regarding the professional domain, there is mounting evidence of the positive effect that the development of ESC has on the performance of leaders. The modern competency movement stems from McClelland (1973), who pointed out the primacy of competencies over traditional measures of intelligence and skills as a predictor of job performance and other outcomes. In the 1980s and 1990s, researchers acknowledged that the vast majority of competencies that predicted effective performance were in the domain of emotional and social behaviors (Boyatzis, 1982; Spencer and Spencer, 1993). All things considered, the choice of ESC seemed to be an ideal competency model for revisiting the methodology in MSF.

## Materials and Methods

### Participants

Participants in our study were enrolled in a leadership development program that has been offered in a leading European business school as a core element of its MBA program since 2001. As part of this development course, full-time MBA students (with previous employment history) participated in 360° assessment of 14 emotional, social and cognitive competencies.

The study is based on a final sample of 887 full time MBA students. Students who did not receive a response from at least three raters in either of the two rater categories –professional and personal– were not considered for the study. The age range of participants was 25–37 (mean = 31.3, *SD* = 2.70); 71% were men, 29% women; 38 countries were represented (highest numbers: United States 17.2%, Spain 9.1%, Germany 9.1%, India, 6.1%) as well as many different educational backgrounds.

All data were collected under the informed consent and followed the ethical guidelines of ESADE University. Participants in this leadership development program always had the option of using the padlock (close option) in case they did not wish to make their data available for research purposes.

### Measures

The assessment tool is the Emotional and Social Competency Inventory—University Edition (ESCI-U), which consists of 70 items^2^ (five per competency) measuring the frequency of behaviors associated with the competencies (Boyatzis and Goleman, 2007). Each item starts with *How often do you/does the target…* and is followed by a behavioral description such as *…understand another person’s feelings?* The questionnaire on the online platform uses an 11-point scale to assess the frequency of each behavior (Batista-Foguet et al., 2009). Scores range from zero (*never*) to 10 (*always*). To avoid biased results, each question also includes the option *Don’t know*, and to avoid inflated correlations among items of the same competency, the 70 items are randomly presented.

Students complete a self-assessment questionnaire and nominate multiple raters, who are then asked to assess the target student using the same questionnaire. Students are asked to indicate to which of the groups and subgroups the raters belonged, professional (supervisors, peers, subordinates and others), or personal (spouses, friends, relatives, classmates, and others). Raters are informed about the purely developmental purpose of the assessment, and confidentiality and anonymity are assured.

### The CFA Model: Dyad Specification

**FIGURE 1:**
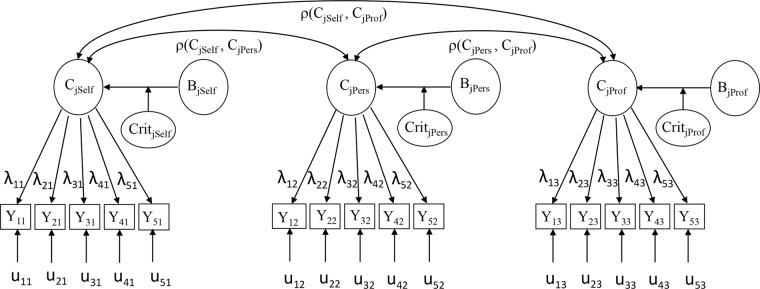
Path diagram of the CFA model which specifies that each dyad (competency-context) has 5 observed indicators (items).

Our approach specifies a CFA model for each competency (Figure 1) with five items per dyad and as many dyads as there are sources in the MSF. Although here may be other forms of interpersonal context that affect perception of raters, to illustrate our methodology we first focused on the two broader perspectives from external raters, personal and professional relationships. Each competency is therefore assessed through three different perspectives: self-assessments (*j* = 1), personal context (*j* = 2), and professional context (*j* = 3), which leads to three dyads per competency. For comparison purposes, we also operationalize the dyads considering the more specific groups (e.g., Friends and Peers) from within each perspective. We therefore substitute in the model specified in Figure 1 the ratings from the wider Professional and Personal categories by the ratings of the more specific Peer and Friend groups. The factor analysis model assumes that the variation in observed scores (items) is due to the variation in the latent factor and in the unique component (u), the latter including specific as well as measurement error variation.

The proposed specification has some similarities to the HCFA used for performance assessment by Scullen et al. (2003), allowing each trait–context combination to be represented by a latent variable. We refer to this combination as the dyad. Notice that since the model from Figure 1 is not identified we decided to merge within the dyad, besides the trait, the effect of the contextual differences in the individual behaviors and in the interaction with the rater criteria and, unlike Scullen et al. (2003), who based their analysis on item parcels (means of multiple items) with two raters per category, we perform the analysis at the item level with at least three raters per category whose assessments are averaged.

### The Response Functions Associated With the Dyads in MSF: Another Perspective on CFA

**FIGURE 2:**
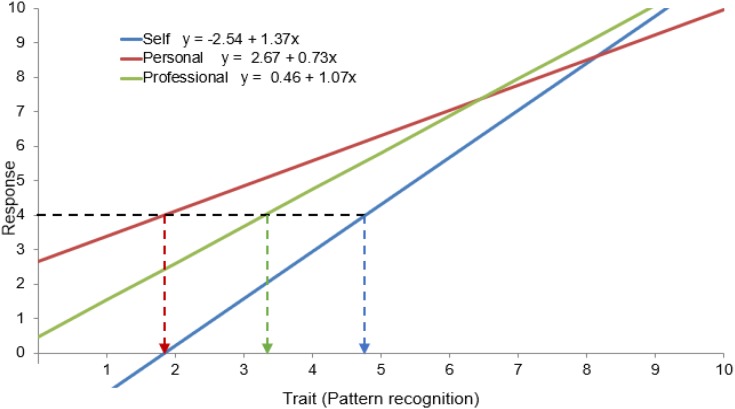
Unstandardized coefficients for pattern recognition, for which metric and scalar invariance are rejected.

The visual representation of response functions (RF) associated with the dyads enables a better understanding of the methodology we propose for MSF. While the factor analysis model usually structures the correlation matrix among observable variables, the response functions displayed in Figure 2 are based on the covariance matrix and specify the relation between observable variables and latent factors (traits), taking into account the structure of the means as well as the covariance structure. Unlike MSPR data analysis, MSF data analysis eventually involves mean comparisons among self and external raters, and therefore, in addition to the covariance structure, the mean structure is also relevant.

The RF help us visualize that diverse assessments across dyads can lead to different RF, which could easily mask differences in the meaning of the underlying characteristic. For illustrative purposes the first item of the competency *pattern recognition* was arbitrarily chosen. For the same observed value (*y* = 4) of the item, differences in the RF of self and the other two sources lead to three different trait values (a bit below 5, slightly below 2, and slightly above 3). In the same way, the same value of the *pattern recognition* trait would lead to different values of the items.

Figure 2 clearly shows that a comparison between sources requires that self and external raters with the same opinion should give the same response. Otherwise, differences in slope (due to different loadings) and intercepts (due to different origins) of their RF would mistakenly be interpreted as real differences in the underlying construct.

In summary, while data analysis to test factor invariance in MSPR is focused on the covariance structure, we propose that data analysis in MSF should focus not only on the covariance structure but also on the mean structure, and to this end testing the invariance of the intercepts (i.e., scalar invariance) is key.

### MSF Measurement Equivalence

Factor equivalence was tested through sequential steps. First, we evaluated the extent to which the five items per competency fit the basic model of Figure 2. *Configural invariance* means that the same single factor model applies to each competency regardless of the source.

Second, we tested for *metric invariance* (or measurement unit equivalence), which also requires that the item loadings (slopes in Figure 2) are the same in the three dyads. The fulfillment of this test allows us to interpret the estimated correlations among the dyads. However, as Byrne et al. (1989) argued, if some of the items (at least two) fulfill these conditions it is sufficient to anchor a common meaning to the factors between groups—the so-called *partial factorial invariance*.

Finally, *strong factorial invariance* (*scalar invariance*) requires that the origins of the measurement scales (intercepts in Figure 2) be fixed on the same point, so that the levels between self and other raters are comparable. This *scalar invariance* is a requirement for comparing factor means between self and other rater groups, and again, this requirement is fulfilled with at least two items (partial) in each dimension.

Previous studies on MSPR, such as Scullen et al. (2003), followed Vandenberg and Lance (2000), in assessing the invariance of the latent structure of job performance ratings across different rater categories. After configural and metric invariance, their third requirement was testing for the equality of the unique variance. As mentioned, in MSF, however, since the aim is to compare self-ratings with means from other rating groups, our third requirement must necessarily be strong scalar invariance (Meredith, 1993) which, as seen in the RF, means testing for equality of intercepts.

### Model Estimation and Fit Diagnosis

Since the distributions for the three dyads deviate from normality, Satorra–Bentler robust Chi-square was considered appropriate as a global goodness-of-fit index for the model. Since this Chi-square test is a test of exact fit, we also used the Root Mean Square Error of Approximation (RMSEA), which is a test of close fit that also takes parsimony into account. Following Credé and Harms (2015) recommendations regarding the global fit for higher-order CFA, besides the two fit indices mentioned above, we also chose the standardized root-mean-square residual (SRMSR), and the comparative fit index (CFI). The latter being the most trustworthy index for nested model comparisons (Cheung and Rensvold, 2002).

However, since it is well known that such global fit indices are based only on statistical significance without taking into consideration the statistical power, they may have significant drawbacks (Saris et al., 2009). In essence, rejection of the model can be due to very small misspecification (actually irrelevant) to which the test is very sensitive (high power) and, non-rejection may happen despite a very large misspecification to which the test is insensitive (low power). We therefore took into consideration the statistical power, which not only depends on the distribution of the data (kurtosis), sample size, model parsimony, the indicators’ reliability, but also on the size of the misspecification and on the size of the incidental parameters (see Saris et al., 2009).

Since our three-dyad model (Figure 1) is not complex, our sample size is large, and reliabilities are relatively high, we are in a high-power situation in which model rejection by a statistical test would not be so relevant. Therefore, in the diagnostic stage, we avoided what Kline (2010) termed “global fit indices tunnel vision,” which might lead us to focus on indices of overall model fit and to ignore more detailed diagnostic indicators. Consequently, we checked the sensitivity of every parameter-modification index jointly with the expected parameter change, as well as the plausibility of its estimated value along with the power of the test and its significance level. Following Saris et al.’s (2009) proposition, we focused on the detection of misspecification errors (column *g* in Table 1) rather than solely on global fit.

**Table 1:** Global fit indices of the configural invariance test of the CFA model as shown in Figure 1 (n = 887 sample).

Competency	ΔSB-χ^2^ (df)	RMSEA	CI_RMSEA_	PCI	CFI	SRMSR	Missp
ESA	157 (87)	0.033	0.025; 0.041	1.00	0.99	0.033	None
IL	242 (87)	0.049	0.042; 0.056	0.08	0.98	0.037	1
ESC	256 (84)	0.052	0.045; 0.060	0.04	0.98	0.034	3
PO	187 (84)	0.040	0.033; 0.048	0.73	0.99	0.042	1
AO	225 (87)	0.046	0.039; 0.054	0.23	0.98	0.037	2
DO	271 (87)	0.053	0.046; 0.053	0.03	0.97	0.039	2
AD	182 (87)	0.038	0.030; 0.046	0.94	0.98	0.034	None
EMP	186 (87)	0.040	0.033; 0.048	0.90	0.98	0.034	1
INF	212 (87)	0.044	0.036; 0.051	0.54	0.97	0.038	1
CFM	808 (87)	0.110	0.099; 0.110	0.00	0.83	0.075	4
OA	200 (87)	0.042	0.034; 0.049	0.75	0.98	0.041	1
TW	200 (87)	0.043	0.035; 0.051	0.21	0.99	0.037	2
ST	221 (87)	0.045	0.038; 0.053	0.50	0.97	0.041	2
PR	386 (87)	0.068	0.061; 0.075	0.00	0.93	0.046	3
	(a)	(b)	(c)	(d)	(e)	(f)	(g)


*(a) Satorra–Bentler Chi square (degrees of freedom) (b) Root Mean Square Error of Approximation (RMSEA), (c) Confidence interval and (d) Probability of close fit for RMSEA (e) Comparative Fit Index (f) Standardized Root mean square residual (g) Detected misspecifications (above Δ = 0.1) for a power > 0.8.*

## Results

### Testing for Model Fit

To properly interpret the results from the confirmatory analysis, goodness-of-fit must first be assessed, and the assessment should be based not only on statistical significance, but also on the power of the global tests and on the model misspecifications (Saris et al., 2009).

Table 1 shows the results (*n* = 887) for the configural invariance test of the three-dyad CFA model as specified in Figure 1. By virtue of the mentioned global fit indices (based just on significance) we would not reject the CFA model. However, we identified some misspecifications within three competencies, ESC, PR and CFM, for which we could find plausible explanations. Regarding PR and CFM, the wording of the five items reflected in fact bi-dimensional factors. Therefore, configural invariance should not be granted for these two competencies. Regarding ESC, the relatively high correlations among residuals of the same items across the three sources could also signal an additional underlying dimension.

Other detected misspecifications (column *g*) were in fact due to having too high statistical power,^3^ and were therefore not included in the model (although their inclusion would have greatly improved the global fit indices). Most of them were residual correlations within a dyad, as a result of two of the items sharing some common wording (specificity). We therefore argue that the results reported in Table 1 support the factorial structure displayed in Figure 1 for all competencies except two (PR and CFM).

### Testing for Metric and Scalar Invariance

Adding the necessary constraints into the configural model for testing metric and scalar invariance leads to nested models, the results of which are displayed in Tables 2, 3. Regarding metric invariance, the constraints – equal loadings across dyads – do not lead to deterioration in the global fit of the model for most of the competencies. Indeed, for this sample, all global fit indices (SB-χ^2^ RMSEA, CFI, SRMSR) and their relevant increments (ΔSB-χ^2^, ∇CFI) meet any significant threshold for all nested models, as seen in Table 2. In fact, for DO and EMP the global fit indices even improve. Again, most of the detected misspecifications (column h) are taken to correspond to either incidental parameters or the test specific sensitivity. None of the detected misspecifications are plausible, except for those detected again in ESC.

**Table 2:** Global fit indices of the metric invariance test and correlations among the three dyads relative to the CFA model as shown in Figure 1.

Competency	ΔSBχ^2^ (Δdf)	RMSEA	CI_RMSEA_	PClos	CFI	SRMSR	r_S-Pe_	r_S-Pr_	r_Pr-Pe_	Missp
ESA	15(8)	0.034	0.026; 0.042	1.00	0.99	0.038	**0.33**	**0.24**	**0.30**	None
IL	11(8)	0.047	0.040; 0.054	0.14	0.98	0.041	**0.31**	**0.34**	**0.36**	1
ESC	18(8)	0.051	0.044; 0.058	0.03	0.98	0.045	**0.33**	**0.32**	**0.40**	3
PO	27(6)	0.042	0.035; 0.050	0.55	0.99	0.051	**0.41**	**0.25**	**0.39**	1
AO	14(8)	0.045	0.038; 0.052	0.25	0.98	0.042	**0.31**	**0.28**	**0.35**	2
DO	-23(8)	0.047	0.040; 0.054	0.30	0.98	0.039	**0.26**	**0.20**	**0.31**	2
AD	37(8)	0.042	0.034; 0.049	0.79	0.97	0.045	**0.22**	**0.27**	**0.36**	2
EMP	-25(8)	0.032	0.024; 0.040	1.00	0.99	0.034	**0.18**	**0.10**	**0.29**	None
INF	10(8)	0.042	0.035; 0.049	0.71	0.97	0.041	**0.33**	**0.26**	**0.31**	2
CFM	–	–	–	–	–	–	–	–	–	–
OA	10(8)	0.040	0.033; 0.048	0.82	0.98	0.045	**0.13**	**0.23**	**0.32**	2
TW	12(8)	0.042	0.034; 0.049	0.29	0.99	0.040	**0.19**	**0.22**	**0.37**	None
ST	10(8)	0.044	0.037; 0.051	0.63	0.97	0.043	**0.27**	**0.20**	**0.30**	2
PR	–	–	–	–	–	–	–	–	–	–
	(a)	(b)	(c)	(d)	(e)	(f)		(g)		(h)


*(n = 887). (a) Satorra–Bentler Chi square (degrees of freedom); (b) Root Mean Square Error of Approximation (RMSEA); (c and d) confidence interval and probability of close fit for RMSEA; (e) Comparative Fit Index; (f) Standardized Root Mean Square Residual; (g) estimated correlations (in bold) among dyads of the same competency; (h) detected misspecifications (above Δ = 0.1) for a power > 0.8.*

**Table 3:** Scalar invariance test.

Competency	ΔSBχ^2^ (Δdf)	ΔRMSEA	CI_RMSEA_	PCI	∇CFI	ΔSRMSR	Missp
ESA	34(6)	0.03	0.031; 0.045	0.98	0.01	0.01	None
IL	11(4)	0.01	0.041; 0.055	0.12	0.00	-0.01	1
ESC	6(4)	0.00	0.044; 0.053	0.06	0.00	0.00	3
PO	17(4)	0.01	0.032; 0.047	0.85	0.00	0.00	1
AO	26(4)	0.00	0.041; 0.055	0.12	0.01	0.00	1
DO	66(4)	0.01	0.048; 0.062	**0.01**	0.02	0.02	3
AD	17(4)	0.00	0.036; 0.043	0.72	0.00	0.00	2
EMP	8(6	0.02	0.025; 0.041	1.0	0.00	0.00	2
INF	42(4)	0.01	0.040; 0.054	0.29	0.01	0	4
CFM	–	–	–	–	–	–	–
OA	107(4)	0.01	0.048; 0.061	**0.01**	0.02	0.00	3
TW	16(6)	0.00	0.035; 0.049	0.32	0.00	0.00	1
ST	2(6)	0.00	0.035; 0.049	0.82	0.00	0.00	2
PR	–	–	–	–	–	–	–
	(a)	(b)	(c)	(d)	(e)	(f)	(g)


*Increments of the global fit indices relative to the metric invariance test, relative to CFA model in Figure 1 (n = 887). (a) Increment in Satorra–Bentler Chi-square (Δdf, difference in degrees of freedom); (b) increment in RMSEA; (c and d) confidence interval and probability of close fit for RMSEA; (e) increment of the Comparative Fit Index; (f) increment of the standardized root mean square residual; (g) detected misspecifications (above Δ = 0.1) for power > 0.8. In bold are the two competences (DO and OA) which rejected Close.*

Configural and metric equivalence are therefore fulfilled in 12 out of 14 competencies, which enables interpretation of the correlations among the three dyads within each of those 12 competencies. The fact that the estimated correlations (column *g*, Table 2) are so far from 1 lead us to draw the following conclusions. First, we conclude that although the items are the same, the context (self or personal/professional) is crucial, as is established by our hypotheses. Second, that the low magnitude of these correlations reflects, as expected, empirically distinct constructs^4^ which constitutes the major justification for conceptualizing the constructs as dyads. And third, that the joint effect of both sources of bias –contextual individual behavior and contextual rater criteria– can be estimated as the magnitude of the attenuation from a correlation among dyads equal to one.

Finally, regarding the scalar invariance test, Table 3 shows that the intercepts’ equality among dyads, required for the comparisons between the means of self and others’ assessments, is rejected for two of the twelve remaining competencies: DO and OA (for which even the close fit, in column d, indicates rejection). We would also reject scalar invariance for ESC, as the detected misspecifications were persistent throughout the three tests. As a consequence, we are only entitled to compare the self-assessment with the mean of personal ratings and with the mean of professional ratings, for 9 out of the 14 competencies. For any of these 9 competencies it is sufficient, for the purpose of mean comparisons, to have only two items that are scalar invariant across dyads (i.e., having partial scalar invariance). In such situations, these would be the only items to be used to assess the gap between external raters and self-evaluations.

Results from the three tests show that the perspective of the different rater groups providing MSF does matter. For five competencies, different rater groups see such different behaviors of the same individual that quantitative comparisons between perspectives could be misleading. In this respect, Toegel and Conger (2003) have already proposed that MSA be more qualitative when used for development, and more quantitative when used for performance. We argue that MSF should ideally include both types of assessment, but for quantitative assessment to be useful and effective, two conditions need to be fulfilled. First, ratings from different perspectives should be comparable (i.e., same response functions), and second, rater groups should be discarded when assessing competencies for which they are not qualified to assess. How to evaluate the second condition (i.e., how pertinent each rater group is for assessing a particular competency) constitutes our third research question.

### Degree of Pertinence of Each Rater Perspective in Assessing a Specific Competency

To answer our third research question, we build on what Atkins and Wood (2002) did in MSPR, by evaluating which of the sources (self, supervisor, peers, or subordinates) provides the most valid assessment of a specific competency. We propose the triangulation of three criteria for evaluating the pertinence of each rater perspective in assessing a specific competence, so that the questionnaires can be tailored to each rater group. This customization should increase not only the relevance of the ratings, but also data quality since shorter questionnaires diminish fatigue and boredom of the respondent. The final purpose is to make the entire 360° feedback process more effective.

The three criteria we propose are the following: (1) the degree of homogeneity of the assessments done by each rater group on each competency, which is assessed through global reliability indices and only applies if the nature of the items is reflective (Jarvis et al., 2003; Bisbe et al., 2007); (2) assessment of the pertinence by field experts based on their substantive knowledge; and (3) the number of *I don’t know* each rater group produces for each competency.

Regarding the homogeneity of assessment on a particular competency (the first criterion), we expected this to be higher for those groups that included less variation of perspectives, thus leading to higher reliability indices. We hypothesized that subcategories (such as peers and friends) would lead to higher consistency in their evaluations than the wider professional or personal perspectives. This hypothesis was tested on a subsample (*n* = 197) consisting of individuals with at least three raters in the same subcategory, such as *friends* from the personal raters, and *peers* from the professional raters.

Since results can only be interpreted after the model fit has been assessed, we first tested the goodness-of-fit of the CFA model from Figure 1, specifying along with the Self, the Peers and Friends dyads instead of the Professional and Personal dyads. Results show much better global fit indices for the subcategories of *friends/peers* than for the broader rater categories. It could be argued that the smaller sample size of the subcategories would lead to less probability of rejecting the model (due to less statistical power). However, the negative effect of the small sample size on the power of the test is counterbalanced by the positive effect of the higher loadings that result from more homogeneous categories.

**FIGURE 3:**
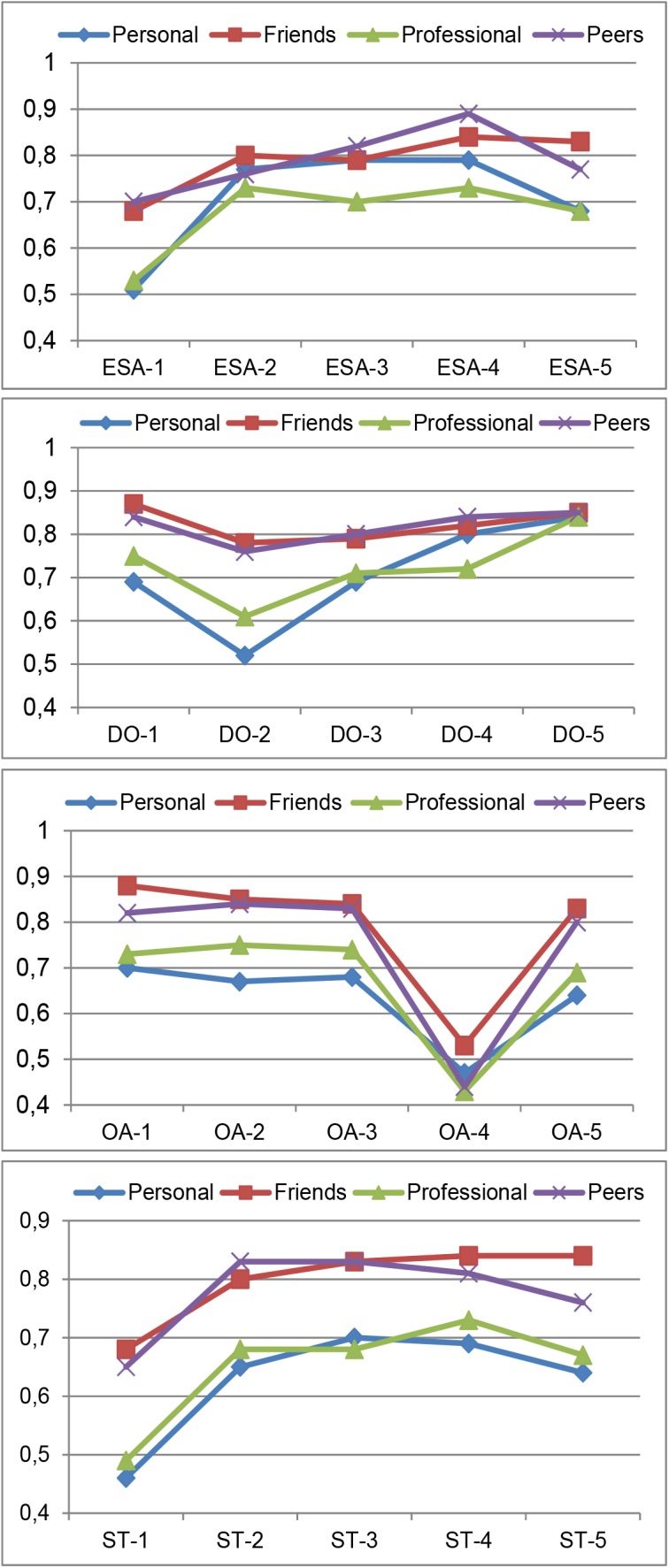
Loading estimates for the 5 items of four competencies: ESA, DO, OA, and ST. The loadings correspond to the Personal, Friend, Professional, and Peer dyads operationalized as in Figure 2, based on the two samples: *n* = 887 (for Personal and Professional) and *n* = 197 (for Friends and Peers).

Indeed, results show that for each competency the loadings from the *friends/peers* rater group are always higher than those from the personal/professional raters. For reasons of space^5^, only reliability comparison profiles of four reflective competencies are shown in Figure 3. Moreover, far fewer misspecification errors were detected in the estimated CFA model for these two subcategories of raters, which helped in obtaining a better model fit.

Reliabilities of the items for each competency are usually computed using Cronbach’s alpha. However, this requires that the items be tau-equivalent, otherwise alpha is biased (Raykov, 1997). The simplest alternative is Heise and Bohrnstedt’s Omega (Heise and Bohrnstedt, 1970), which only requires a unidimensional factor analysis model fitted to the (in our case five) items of each competency. Table 4 indicates (bold type) the appropriate reliability index depending on the tau-equivalence requirement. Results show that all global reliability estimates are much higher for the more homogeneous subcategories than for the more general group categories.

**Table 4:** Comparison of the alpha and omega global reliability measures of the three dyads as shown in Figure 2.

	Personal	Friend	Professional	Peer
	raters	raters	raters	raters
**Competency**	***n* = 887**	***n* = 197**	***n* = 887**	***n* = 197**
ESA	**0.902**	**0.937**	**0.875**	**0.938**
IL	0.872	0.915	0.880	0.927
ESC	0.887	0.927	0.891	0.953
PO	0.850	0.885	0.849	0.885
AO	**0.851**	**0.893**	**0.867**	**0.912**
DO	**0.901**	**0.939**	**0.889**	**0.936**
AD	**0.791**	**0.910**	**0.843**	**0.920**
EMP	0.871	0.907	0.858	0.917
INF	0.741	0.880	0.741	0.862
CFM (2 factors)	0.792	0.853	0.789	0.850
OA	0.769	0.861	0.791	0.881
TW	0.870	0.838	0.898	0.849
ST	**0.879**	**0.920**	**0.791**	**0.905**
PR (2 factors)	0.763	0.875	0.759	0.853


*Omega (in bold) is used when tau-equivalence assumption is not fulfilled. CFM and PR are not applicable since they are not unidimensional.*

Given that homogeneity of assessments is highest at the lower levels of aggregation (i.e., ratings by subcategories instead of by broader categories), analysis of the degree of rater pertinence was conducted at the most detailed level of rater groups. Hence, reliabilities and loadings, as a first criterion for triangulation, were computed at that level.

With respect to substantive knowledge (the second criterion for triangulation), three field experts independently assessed the degree of pertinence (on a scale from 1 to 10) for each rater group to evaluate each competency. When pertinence was unanimously assessed below 5, the related competency was a candidate to be excluded from the survey specific to that rater group. Disagreements were discussed, and new candidates were added only if consensus among the three experts was reached.

The third criterion for triangulation refers to the difficulty that a rater group has in assessing the competency. Partial or no knowledge of a competency was analyzed by computing the percentage of people, within each rater group, who answered with at least one *I don’t know*. Not being able to assess one item was interpreted as an indication of the rater’s difficulty in assessing the competency. When the percentage of people with partial or no knowledge was greater than 20%, the competency in question was a candidate to be excluded from the survey specific to that rater group.

None of the few missing values were imputed as they could be indicative of the raters’ poor knowledge about the ratee, and as such, constituted a vital piece of information to be kept for this triangulation analysis.

Table 5 shows the results of the triangulation analysis. The presence of all three numbers (1, 2, 3) for a given competency-subcategory combination, indicates that none of the three criteria were fulfilled, and therefore the subcategory is not considered pertinent for properly assessing that particular competency. Results show that no subcategory seems pertinent for properly assessing influence (INF), system thinking (ST), and pattern recognition (PR). Additionally, neither of the professional subcategories nor the classmates seem pertinent for properly assessing emotional self-awareness (ESA). Also, partners, friends and family do not seem pertinent for assessing adaptability (AD) or organizational awareness (OA). We therefore propose that the survey be customized to each rater subcategory by eliminating those competencies that cannot be properly assessed.

**Table 5:** Criteria fulfilled by each rater group on lack of pertinence to assess a competence.

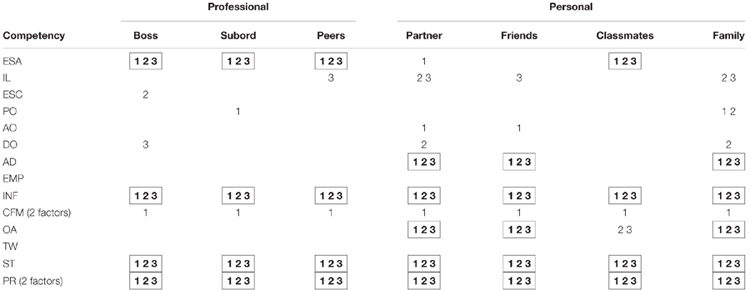

*1 = homogeneity of assessments (reliabilities); 2 = substantive knowledge; 3 = % of I don’t know. Results in a square indicate that the three criteria for exclusion hold, and consequently the competency is proposed to be withdrawn from the survey for the corresponding rater group.*

For the competencies that fulfill the triangulation criteria, the analysis clearly shows that pertinence is a matter of degree: some subcategories of raters are better suited than others in assessing competencies. For example, inspirational leadership (IL) seems to be best evaluated by bosses and subordinates, whereas emotional self-control (ESC) and positive outlook (PO) appear to be best evaluated by partners, friends and classmates.

To sum up, the triangulation analysis allowed us to tailor the initial survey consisting of 14 competencies, to two significantly shorter surveys. One with 10 competencies for the professional raters and classmates, and another with 9 competencies for the rest of the personal sub-groups.

## Discussion and Conclusion

Multisource assessment can be used for either performance management (MSPR) or development (MSF) purposes. When used for performance management, the aim is that all raters evaluate the same performance dimensions, evaluations that are usually considered for administrative decisions, such as compensation or promotion. In MSPR, convergence of individual ratings is therefore desirable. MSF differs from MSPR in two fundamental aspects. First, as MSF is used for development purposes, obtaining feedback (in aggregated ratings) from a multitude of perspectives helps the ratee to obtain a more comprehensive assessment of the self. Second, self-other rating comparisons are central to the MSF process as they help participants to enhance their self-awareness and to make better choices when designing the development plans (Penny, 2001).

There are many studies dealing with the data analysis methodology in MSA, but they mostly concern the MSPR framework. We contribute to the literature of MSA by proposing an adaptation of the data analysis methodology for MSF that takes into account the idiosyncrasies of the development approach, which are primarily the multiplicity of contexts and number of raters, and the relevance of the gap for development purposes.

First, we propose conceptualizing the *dyad* that represents the binomial competency-context as a trait, and to specify an *aggregate* second-order factor model to structure the interrelationship among dyads within each competency. This model better represents the complementary evaluations from the different rater groups. Unlike in MSPR which specifies a latent second HCFA (Law et al., 1998; Wong et al., 2008), in MSF we should never look for evidence of convergent validity. This model specification, however, leads to a more complex estimation and model testing.

Facteau and Craig (2001) provides an illustrative example of the opposite results achieved in a MSPR context. Testing measurement invariance through CFA and item response theory, showed that results across self, peer, supervisor, and subordinate raters were invariant across these rater groups. However, (2005) in a Meta-Analysis integrating 90 years of empirical studies shown that in a great extent intercorrelations among rated job performance dimensions were substantially inflated by differential halo effects from the rater categories.

In our MSF context, the *aggregate* nature of the multidimensional dyad model (i.e., the second order factor) is evinced by the very low correlations among dyads within the competency, which is an indication that external raters and self-assessment probably have different criteria or evaluate different nuances of the same competency. This finding justifies the dyads conceptualization.

Second, since raters can never be fully crossed with ratees in MSF, aggregated ratings must be considered for data analysis, and the eventual comparison between rater group means and self-assessment is critical. Therefore, we propose following the guidelines from Vandenberg and Lance (2000) usually used to test factor invariance in MSPR, but replacing their third test (equivalence of unique variances) with Meredith’s (1993) scalar invariance test. The response functions we used to illustrate the CFA model, which take into account the structure of the means as well as the covariance structure, helped in visualizing how comparing means requires the invariance of the origins (scalar invariance) of the measurement scales used by the self and all other rater groups.

Third, we propose a novel way of customizing the surveys to each rater group based on the triangulation of three criteria. Individually, each criterion is known to determine whether a rater group is appropriate for assessing a particular competency or not. First, we should pay attention to the reliability indices for each competency, as an indicator of measurement quality (London and Smither, 1995; Conway and Huffcutt, 1997). For a given competency, the highest reliability indices should come from the most pertinent groups. Results also show that the higher the rater homogeneity (more specific subcategories of raters), the higher the reliability for measuring specific competencies. Second, as suggested in previous MSPR studies such as Borman’s (1974), we should use substantive knowledge to decide which contexts were best suited for evaluating which competencies, on the basis of the number of opportunities to observe the specific competency being assessed. Third, we should consider the rater knowledge about the ratee (Farr and Newman, 2001), which in our MSF data can be easily identified as the number of *I don’t know* responses that each context provides for the same competency.

In the framework of our study, the survey customization involved the elimination of three competences (INF, ST, and PR) across all subcategories. Our interpretation of these results is that in some contexts raters did not have many good opportunities for observing the related behaviors due to the young age of the ratees (MBA participants), therefore claiming that they do not have the knowledge to evaluate (i.e., high % of contexts with at least one *I don’t know*), and when they venture to do it, their observations are unreliable.

Additionally, the triangulation analysis for rater pertinence confirmed our conjecture that some specific subcategories may not be well suited for assessing some of the competencies. In this respect, only partners, friends and family seem to be able to properly assess emotional self-awareness (ESA), a competency that is more likely to manifest in personal domains. In contrast, only professional contexts and classmates seem pertinent to properly assess adaptability (AD) and organizational awareness (OA), competencies that measure behaviors which are mostly work specific (e.g., *adapt overall strategy, goals, or projects to fit the situation*).

However, triangulation results seemed inconclusive for a few competency-subgroup combinations, revealing two different situations that deserve further analysis. The first situation refers to some combinations in which the degree of knowledge (percentage of contexts with at least one *I don’t know*) was low, while the reliabilities were high, as for example the case of developing others (DO) assessed by the boss. Since not all young MBA students led teams during their professional experience, some bosses were not able to evaluate all the items corresponding to that competency, but for those bosses who were, the assessment was highly reliable. We therefore conclude that, in this situation, the subgroup should be considered pertinent for assessing the competency.

The second situation refers to those combinations which showed a high degree of rater knowledge but with low reliability, as was the case of achievement orientation (AO) as assessed by the partner or friends. A possible explanation could be that the ratee is likely to manifest this competency in both the personal and professional domains but in very different ways. Some raters may differ in choosing the context in which to base the assessment, thus leading to low correlations among ratings (and thus low reliabilities).

On the whole, this triangulation analysis allows us to customize the survey by incorporating, for each context, only the set of competencies most suitable to be assessed by that rater group in question. With shorter, customized surveys, the validity and the relevance of the feedback should increase, and consequently, the 360° feedback process should help participants to design more appropriate development goals and plans.

Although the methodology presented in this paper has been developed in a study that conceptualizes context, as the type of relationship (personal versus professional) between raters and the ratee, this methodology can be equally applicable to studies using many other contextual dimensions prone to generating rating discrepancies in MSF. One example could be studies in which context is determined by the quality of the relationship (convivial and cooperative versus competitive), since having a shared vision or having compassion for the ratee (Boyatzis, 2018) could affect how raters evaluate or interpret the ratee’s emotions and behavior. Another example could be studies that conceptualize context in terms of cultural dimensions with regards to relationships as discussed by Hofstede (1980). Specifically, research has revealed that power distance influences the perception of three leadership skills: decision-making, leading employees and composure, and that as a consequence such cultural context generates MSF-rating discrepancies (Eckert et al., 2010), which makes it another ideal case for MSF analysis using our new methodology. Beyond power distance, other cultural dimensions are also likely to generate rating differences. In a hierarchical and masculine oriented culture for example (such as in Latin America), raters might be reluctant to evaluate people with higher status other than being positive or obsequious, in comparison to evaluations of raters that come from more egalitarian cultures (such as Germany, Finland, or the Scandinavian countries). There may also be cultural differences stemming from the nature of industry, as raters with a strong history in the high tech, fast moving, adaptive and diverse organizational cultures, are likely to assess competencies differently from raters that come from a more stable manufacturing culture. In conclusion, awareness of the role of the context on MSF together with the use of our new method will help the design of MSF surveys to better capture the nuances of the context concerning the different competencies being assessed.

### Contributions

With our present study we aim at contributing to both methodology and practice. First and foremost, we contribute to the literature of MSA by presenting a new methodology for data analysis in MSF. Our proposed methodology is based on the notion that in MSF, in contrast to MSPR, convergence of assessments should not be expected. In MSF, each rater group offers a complementary perspective that helps the ratee form a more complete picture of the self. This notion leads us to propose a data analysis that follows a three-step approach: (1) to conceptualize *competency-context dyad* as the trait, which we propose should be the first order factor in a multidimensional aggregate (instead of latent) second-order factor model; (2) to substitute the third requirement of MSPR by the test for scalar invariance, a requirement for mean comparisons; and (3) to customize the survey according to context pertinence using the triangulation of three criteria (reliability, substantive knowledge based on field experts, and % of don’t knows) with the aim of improving the data quality and the usefulness of the feedback.

This study has also some important implications for practice. When recipients of MSF (ratees, or coaches/HR practitioners assisting the ratees during the MSF process) evaluate the feedback, they should refrain from analyzing average scores in broad categories. Different perspectives, as the blind men experienced when touching the elephant, grasp different aspects of reality, and therefore averaging scores could be meaningless, or at least misleading. Recipients of the feedback should look at aggregated scores from raters coming from homogeneous sub-groups, such as bosses, friends or peers. And only when strong factor invariance is fulfilled can mean comparisons between groups and the self be interpreted and considered for an eventual (and appropriate) development plan.

Finally, the proposed triangulation for tailoring the surveys aims to assist practitioners in deciding what competencies are suitable for each context to assess. By making the surveys shorter, the response rate as well as the quality of the assessment are likely to increase. This will lead the ratee to perceive the feedback as more accurate and therefore more useful, especially the feedback from the groups to whom the ratee’s behaviors have greater exposure. For instance, in MSPR studies, Lewin and Zwany (1976) and Luthans et al. (1988) reported that, on the whole, peers were the most accurate observers of a person’s behavior. This could be explained by the ecological fact that subjects spend more time with peers than with other subcategories of raters. The more accurate the feedback is perceived, the harder it is to discount as not useful (Brett and Atwater, 2001).

### Limitations and Future Research

Our proposed data analysis approach is intended to be applicable to any MSF whatever the competency measurement instrument is used. We acknowledge that results and conclusions from our study are context-based (i.e., they depend on the choice of competencies in MSF, their particular operationalization, and the specific sample used). The purpose of the study was not to provide construct validity or external validity of a specific MSF measurement tool, but to illustrate our proposed methodology and to show how this methodology can lead to an improvement in data quality and usefulness of the MSF process.

However, it may be worth exploring how results and conclusions vary in different MSF contexts. Our study was based on the assessment of emotional intelligence competencies, which are likely to manifest in a great variety of contexts. However, the items for some competencies measured behaviors that are typically work specific, which resulted in reliabilities being lower for the personal contexts than for the professional contexts, a fact that had an impact on the degree of survey customization. Further research should explore if framing the context in which these items should be assessed may increase reliabilities and therefore the validity of the feedback.

Additionally, some sub-categories of raters claimed partial or no knowledge in assessing some of the competencies, a fact that we attribute to the young age of the ratees (MBA participants) with a limited work experience. It would be interesting to study how raters respond in a study with ratees of a higher age, such as participants of leadership programs in executive education, and how the customization of the surveys differs from that in our present study.

Finally, it is also quite possible that some of the raters from the professional and personal environments may have been lenient in their evaluations because the surveys had been sent through the business school to which the ratee has just been accepted. Hence, social interaction threats are likely to affect the construct validity inferences made from the evaluations provided. All things considered, survey customization merits further study in different scenarios.

Organizations have been increasingly moving from using MSA exclusively as a tool for performance (MSPR), toward using it also for development (MSF). Some organizations and academics have even used the term *performance development* (Clayton and Ayres, 1996; Conway, 2000; Scullen et al., 2003) to reflect the dual purpose of performance management and employee development. However, it is important to acknowledge that the change in purpose implies a change of paradigm. When MSA is used for performance management purposes, convergence of ratings is key. However, when MSA is used for development purposes, organizations should understand that differences in feedback are the cornerstone of the MSF processes. Generating multiple feedback that is diverse and relevant to each context is necessary to create a faithful and comprehensive image of one’s self (i.e., one’s strengths and weaknesses).

However, for feedback differences to be meaningful, it is crucial to check that ratings can actually be compared and that raters are qualified to assess each competency. Scholars doing research in MSF now have a path for designing more useful and effective MSF tools. Organizations engaging in development programs using MSF have an opportunity to seek collaboration with these scholars so that they can help with the survey customization, and with a proper analysis of the feedback data by ensuring that strong factor invariance holds before interpreting the gaps. Many resources and effort are invested in those programs, and practitioners should therefore be concerned about maximizing program effectiveness and helping participants make the right choices when defining goals and development plans. We encourage both scholars and practitioners to work closer together in incorporating our methodology in existing MSF processes, and in further exploring the impact it has on making talent development programs more effective.

## Ethics Statement

This study was carried out in accordance with the guidelines regarding the Use of Human Subjects in Research issued by the ESADE Research Ethics Committee, affiliated to the Ramon Llull University of Barcelona. The study has been reviewed and approved by the ESADE Research Ethics Committee. Written informed consent was obtained from all research participants. A copy of the Application Form for Ethical Review (Approval Number 014/2017) is available at request to the principal author.

## Author Contributions

Since the paper is mostly methodological WS worked closely with JMB-F since the proposal of the RQ and derived hypotheses. The paper is based on ICT, therefore REB who established this theory played a vital contribution in framing and revising the whole research. The research design and data collection has been the responsibility of RS who also provided the basis for the Ethics’ fulfillment. Finally, FVM not only made a critical revision of every step in the research process and of the whole draft, but also had the initiative to extend the paper proposing the application of the methodology proposed for customizing the survey to each source. This is a relevant intellectual contribution which covers the third section of the paper. JMB-F found the gap in the MSF vs. MSPR, proposed the RQs with REB, proposed the RD with RS, and the whole data analysis with WS. JMB-F wrote the first and last drafts.

## Conflict of Interest Statement

The authors declare that the research was conducted in the absence of any commercial or financial relationships that could be construed as a potential conflict of interest.

## Footnotes

1 Our approach to assess individual competencies for development is to gather information not only from the professional but also from the personal environment –Partner, relatives, and friends.

2 The competencies included in ESCI-U are: emotional self-awareness (ESA), emotional self-control (ESC), achievement orientation (AO), adaptability (AD), positive outlook (PO), empathy (EM), organizational awareness (OA), conflict management (CFM), developing others (DO), influence (IN), inspirational leadership (IL), teamwork (TW), systems thinking (ST), and pattern recognition (PR).

3 When the size of any misspecification is negligible (i.e., its *standardized expected parameter change* falls below 0.15) and leads to a great Chi-square increment, this reveals a situation of excessive statistical power (Saris et al., 1987, 2009).

4 Notice that although the assessments from these three sources are expressed in the same way (metric invariance) they clearly provide different opinions. As mentioned, this is due to contextual differences. The behavior observed by the raters is different due to either the manifestation of *multiple/possible selves* by the ratee (i.e., the ratee’s behavior is contingent to the context), or the raters’ criterion for evaluating that behavior is also different, or both.

5 All input matrices are available on request from the first author.
